# Frequency of Fabry disease in male and female haemodialysis patients in Spain

**DOI:** 10.1186/1471-2350-11-19

**Published:** 2010-02-01

**Authors:** Paulo Gaspar, Julio Herrera, Daniel Rodrigues, Sebastián Cerezo, Rodrigo Delgado, Carlos F Andrade, Ramón Forascepi, Juan Macias, Maria D del Pino, Maria D Prados, Pilar R de Alegria, Gerardo Torres, Pedro Vidau, Maria C Sá-Miranda

**Affiliations:** 1 Unidade de Biologia do Lisossoma e do Peroxissoma, Instituto de Biologia Molecular e Celular, Universidade do Porto, Porto, Portugal; 2 Servicio de Nefrologia, Hospital Universitario Central de Asturias, Oviedo, Spain; 3 Servicio de Nefrologia, Hospital Clínico San Cecilio, Granada, Spain; 4 Servicio de Nefrologia, Hospital Virgen del Rocío, Sevilla, Spain; 5 Servicio de Nefrologia, Hospital de Cabueñes, Gijon, Spain; 6 Servicio de Nefrologia, Hospital Clínico de Salamanca, Salamanca, Spain; 7 Servicio de Nefrologia, Hospital Torrecárdenas, Almeria, Spain; 8 Servicio de Nefrologia, Hospital de la Cruz Roja de Oviedo, Oviedo, Spain; 9 Servicio de Nefrologia, Hospital General Yagüe, Burgos, Spain

## Abstract

**Background:**

Fabry disease (FD), an X-linked lysosomal storage disorder, is caused by a reduced activity of the lysosomal enzyme α-galactosidase A. The disorder ultimately leads to organ damage (including renal failure) in males and females. However, heterozygous females usually present a milder phenotype with a later onset and a slower progression.

**Methods:**

A combined enzymatic and genetic strategy was used, measuring the activity of α-galactosidase A and genotyping the α-galactosidase A gene (*GLA*) in dried blood samples (DBS) of 911 patients undergoing haemodialysis in centers across Spain.

**Results:**

*GLA *alterations were found in seven unrelated patients (4 males and 3 females). Two novel mutations (p.Gly346AlafsX347 and p.Val199GlyfsX203) were identified as well as a previously described mutation, R118C. The R118C mutation was present in 60% of unrelated patients with *GLA *causal mutations. The D313Y alteration, considered by some authors as a pseudo-deficiency allele, was also found in two out of seven patients.

**Conclusions:**

Excluding the controversial D313Y alteration, FD presents a frequency of one in 182 individuals (0.55%) within this population of males and females undergoing haemodialysis. Moreover, our findings suggest that a number of patients with unexplained and atypical symptoms of renal disease may have FD. Screening programmes for FD in populations of individuals presenting severe kidney dysfunction, cardiac alterations or cerebrovascular disease may lead to the diagnosis of FD in those patients, the study of their families and eventually the implementation of a specific therapy.

## Background

Fabry disease (MIM 301500) is an X-linked lysosomal storage disorder resulting from mutations in the α-galactosidase A gene (*GLA*), leading to a reduction in the activity of the lysosomal enzyme α-galactosidase A (α-Gal A) [1]. This enzyme defect generates progressive accumulation of globotriaosylceramide (Gb_3_) and related glycosphingolipids, primarily in the vascular endothelium leading to renal failure, cardiac and cerebrovascular disease [1,2].

In general, males with a deficient activity of α-Gal A exhibit the classical FD phenotype with angiokeratomas, acroparesthesias, hypohidrosis, and corneal and lenticular opacities, with onset occurring in childhood. Heterozygous females usually present a milder phenotype, showing a slower progression. However the clinical manifestations of FD in females are highly heterogeneous due to the random X-chromosome inactivation [3]. Therefore, some heterozygous females develop severe manifestations of FD including renal disorder [4].

Clinical variants of FD in male patients with residual activity of α-Gal A have been described. These patients show milder phenotypes and a later onset than those presenting classical symptoms of the disease. Patients with the cardiac variant of FD usually develop clinical symptoms in the sixth decade. These patients present left ventricular hypertrophy (LVH) and/or cardiomyopathy [5-8], and may also develop some degree of proteinuria and renal insufficiency [9]. Patients with the 'renal variant' of FD may present with proteinuria at an early stage, which can eventually progress to end-stage renal failure, being one of the major causes of morbidity and mortality among patients with classic FD [9]. The prevalence of FD has been estimated to have a range between one in 117 000 births and one in 40 000 males [1,10,11], but the precise prevalence is unknown. An accurate estimation of its epidemiology is difficult to do because FD is clinically very heterogeneous and its early classic manifestations tend to be non-specific and often unrecognized. Patients are therefore frequently misdiagnosed, or not diagnosed until late in life [1,12]. Recently a newborn screening showed an incidence of one in 3100 live-newborns, accordingly with this study the later-onset forms of FD present a surprisingly high incidence [13]. This finding is confirmed by recent screening studies of patients undergoing haemodialysis which indicated that the number of patients with unrecognized FD is much greater than the one reported in the dialysis registry data from the USA and Europe [14-16]. Diagnosis of FD (e.g. through screening) is important since it allows the treatment with a specific therapy of affected patients as well as the identification of asymptomatic affected relatives and genetic counselling for couples at risk.

The frequency of FD in Spanish patients undergoing haemodialysis has not been reported to date. The aim of this study was to identify FD patients within male and female patients undergoing haemodialysis. Reduced α-Gal A activity was used as a biochemical marker of FD and to select patients for further studies by genotype analysis. In this study, we report for the first time the frequency of FD in a Spanish population of individuals in haemodialysis.

## Results

### Patients

All the patients (males and females) undergoing haemodialysis and that signed the informed consent were included in the screening. Data were available for 911 individuals (543 males and 368 females). The two groups did not differ significantly in age, being the mean age of 66.2 (range 20.7 - 91.1) and 66.9 (20.2 - 90.4) for males and female patients, respectively.

### α-Gal A activity and genotype analysis

**Figure 1:**
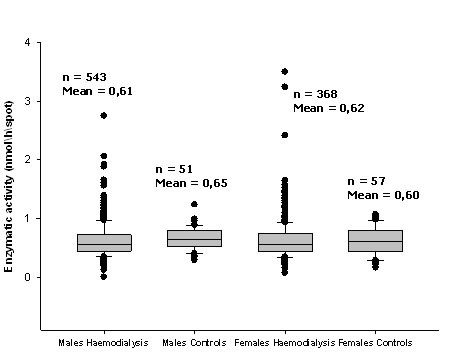
**α-galactosidase A activity in control samples and in a haemodialysis population with both genders**.

The mean value for α-Gal A activity in the 911 DBS samples was 0.625 ± 0.326 nmol/h/spot, similar to the mean value of 0.620 nmol/h/spot of the reference values. Thirty individuals (5.5%), out of the 543 males presented levels of α-Gal A activity below the minimum control range, which corresponded to 48% of the control mean (0.3 nmol/h/spot; Figure 1). One hundred and twenty five females (34%) out of 368 females screened present enzymatic activities below the previously defined cut-off, which corresponded to 80% of the control mean (0.5 nmol/h/spot; Figure 1). In this study, it can not be completely excluded that some heterozygous could remain unidentified since genotyping is the most reliable method to identify FD among females. The used cut-off was selected on the basis of previous experience in diagnosis of female Fabry patients, which usually present activities lower than 60% the control mean. The lowest values obtained for α-Gal A activity was 0.001 nmol/h/spot in male patients, and 0.15 nmol/h/spot in female patients.

**Figure 2:**
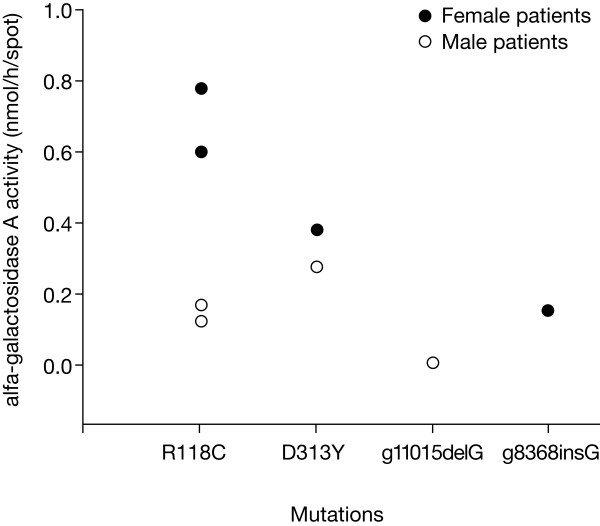
**Activity of α-galactosidase A in patients with FD with different *GLA *mutations**.

Denaturing high-performance liquid chromatography (DHPLC) analysis was performed in the 155 samples from male and female individuals showing α-Gal A activity below the pre-defined minimum. DNA alterations were found in 48 of these samples, from which 40 were polymorphisms or sequence variants (data not shown). Gene amplification and sequencing of single-stranded genomic DNA from the remaining eight samples led to the identification of two novel mutations (p.Gly346AlafsX347 and p.Val199GlyfsX203), and two previously described alterations, the R118C mutation and the D313Y alteration [13,17-21]. (Table1 and Figure 2). The α-Gal A enzymatic activity in the eight patients with FD identified ranged between 0.001 and 0.292 nmol/h/spot in males and from 0.153 to 0.770 nmol/h/spot in females. Whenever the leukocytes from the identified FD patients were available, the α-gal A activity was determined in these cells and the results obtained confirmed the values obtained in DBS. (Table 1)

**Table 1:** Characteristics of patients identified in the screening

Patient	Characteristics	Mutation	Sex	Age, years	α-Gal A activity on Leukocytes, nmol/h/mg of protein	α-Gal A activity on DBS, nmol/h/spot	α-Gal A mutation	
							
							Genomic Reference Sequence (X14448)	Coding DNA Reference Sequence (X14448)
1	Angiokeratoma; fever episodes; hypohidrosis; gastrointestinal problems; proteinuria; progressive hearing loss; valvular dysfunction and arrhythmias; stroke at 71 years; Mother died due to a Transient Ischemic Attack (TIA).	R118C	Male	72	20 (35% of control mean)	0.164 (26% of control mean)	g.5251 C>T	c.352 C>T
2	Abdominal pain; nephropathy; left nephrectomy; cataracts; progressive loss of vision. Sister diagnosed with FD. Presents Front temporal sub cortical and cortical atrophy; periventricular hypodensity of white matter	R118C	Male	83	n.a.	0.122 (20% of control mean)	g.5251 C>T	c.352 C>T
3	End-Stage Renal Disease (ESRD), LHV, valvular dysfunction	D313Y	Female	80	28 (49% of control mean)	0.371 (59% of control mean)	g.10645 G>T	c.937 C>T
4*	Proteinuria glomerulosclerosis and hyalosis in kidney biopsy; kidney transplantation failure; HBP; Human Immunodeficiency Virus (HIV); An uncle and a cousin with kidney dysfunction. Another cousin with hypoacusia	R118C	Female	47	45 (79% of control mean)	0.592 (94% of control mean)	g.5251 C>T	c.352 C>T
5*	Sister of patient 4 also identified as an index patient. LVH in dilatation phase and multi-valvular disease; HBP. Pigmentary macular edema; Cirrhosis, HIV; Son and sister with FD; Both with proteinuria.	R118C	Female	50	34 (60% of control mean)	0.770 (123% of control mean)	g.5251 C>T	c.352 C>T
6	Angiokeratoma; acroparesthesia; fever episodes; abdominal pain; nephropathy; policysts; LVH, auricular fibrillation; acute myocardial infarction. Two daughters diagnosed with FD	p.Val199GlyfsX203	Female	58	n.a	0.153 (24% of control mean)	g.8368_8369 insG	c.595_596 ins G
7	Nephropathy; Psychological problems; kidney transplant, TIA, valvular dysfunction, arrhythmias patient refused to be studied. Relative diagnosed with FD	p.Gly346AlafsX347	Male	54	n.a.	0.001 (0% of control mean)	g.11016delG	c.1037 delG
8	Dyspnea, palpitations, proteinuria, kidney transplant, LHV, valvular dysfunction, arrhythmias.	D313Y	Male	74	22 (39% of control mean)	0.292 (46% of control mean)	g.10645 G>T	c.937 C>T

Control Values DBS - Mean -> 0.62 nmol\h\spot; Minimum -> 0.3 nmol\h\spot

Leucocytes - Mean -> 57 nmol\h\mg of protein; Minimum -> 28 nmol\h\mg of protein

() - Percentage of the mean value obtained in controls and patients, respectively;

**n.a. **- not available; * - sisters

The two novel mutations identified, in patient #7 (Table 1), a guanine deletion produced a premature stop codon instead of transcription and translation to leucine. In patient #6, p.Val199GlyfsX203 in exon 4 also caused a frameshift and the appearance of a premature stop codon at amino acid position 203 instead of a glutamic acid. The two novel mutations were undetected in 180 X-chromosomes from unaffected individuals (60 males and 60 females control samples), which indicated that they were not common sequence variants or polymorphisms. The two novel mutations lead to a stop codon, and therefore to the truncation of the resulting protein product. As expected, like in other truncated proteins, no functional enzyme was found, as observed by the lack of enzyme activity (data not shown). Patients 3 and 8 presented the D313Y alteration, the so called 'pseudo-deficiency allele' [19,22]. The previously described *GLA *R118C mutation [13,21] was found in four patients (#1, #2, #4 and #5) (Table 1) following DHPLC and sequencing analysis. Although patients #4 and #5 were identified independently, they were found to be sisters. The residual α-Gal A activity range between 0.122-0.164 nmo1/h/hspot in hemizygous FD patients with the R118C mutation. This C-T substitution in codon 118, results in the replacement of the strongly basic amino acid arginine by the neutral, potentially sulphydryl-binding, cysteine. The residual α-Gal A activity in hemizygous individuals presenting the R118C or the D313Y was 22% and 40% of the control mean, respectively. These results were confirmed in leukocytes and plasma of patient #1 (R118C) which showed an enzymatic activity corresponding to 35% and 67% of the control mean in leukocytes and plasma, respectively, and of patient #8 ( D313Y) which presented an activity corresponding to 39% and 29% of control mean, respectively (see Table1).

**Figure 3:**
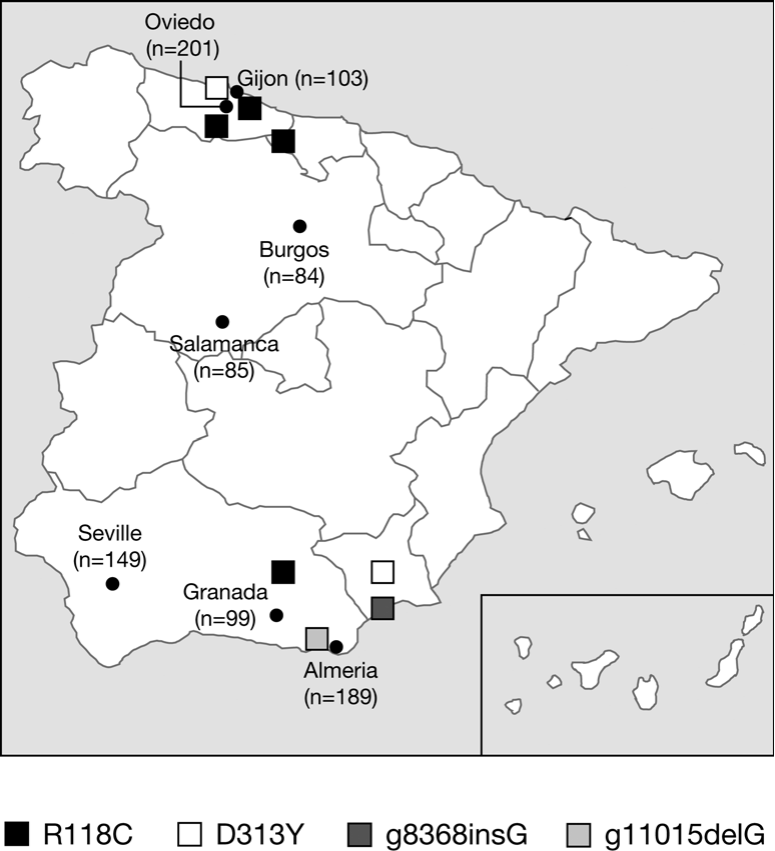
**Location of participating centres and of patients with mutations that cause FD**.

In order to ascertain if the haemodialysis patients with FD mutations were from a particular region of Spain, they were classified according to their geographical area (Figure 3). This showed a random, non-clustered distribution of several mutations. Distribution of the two frequent alterations, R118C and D313Y, was spread across Spain, suggesting that their high frequency was not due to founder effects. Moreover the R118C was not found in 240 alleles from healthy controls, ruling out the possibility of being a polymorphism.

## Discussion

The classic clinical spectrum of FD has been extended to include a large diversity of symptoms. Patients have been described with symptoms restricted to the heart and/or kidney, the so-called renal and cardiac variants [1,2,5-8]. The renal variant of FD was first described by Nakao et al. [14] following the screening of patients undergoing dialysis in Kyushu Island, Japan. The frequency of the renal variant in that study was one in 87 (1.2%) males. In this study, we found seven unrelated individuals with *GLA *alterations, five with FD causal mutations (three male and two females) and two presenting the "pseudo-allele" D313Y [19,22] among the 911 patients undergoing haemodialysis. An overall prevalence of one in 182 (0.55%) was estimated, excluding the two individuals with the D313Y allele, one in 181 among males (0.55%) and one in 184 among females (0.54%). Although the overall prevalence in our study is slightly lower than the frequency of the renal variant of FD found in the Japanese study (1.2%) [14], is significantly higher than the prevalence reported by Linthorts G. et al [23] in a very recent review of all published screening studies carried out in haemodialysis individuals. According to this overview a mean prevalence of 0.33% was found in the study of a total of 7182 males screened in 12 independent studies and a prevalence of 0.10% in the screening of a total of 4179 females in 6 studies. In our study we found a similar prevalence of FD in males (0.55%) and females (0.54%), being the obtained values 1,6 and 5,4 times higher for males and females, respectively than the prevalence reported in literature [23]. These findings suggest that the combined biochemical and genetic strategy used in our screening is more efficient in the identification of FD patients, particularly in the case of heterozygous [23].

All except two of the patients with FD identified in this study had missense mutations, resulting in residual activity of α-Gal A. This activity explains why these patients did not show the early classic manifestations of FD. Two of the mutations identified had not been reported previously. The D313Y alteration lead to reduced activity of α-Gal A and has been associated with FD presenting with cardiovascular symptoms [20]. In contrast to previously described, where association between D313Y and other *GLA *mutations was reported [24,22], in our study the D313Y was the only *GLA *alteration present in the mutated allele. The R118C mutation is also associated with an amino acid substitution and residual activity of α-Gal A. Molecular modeling of this mutation indicated that the substituted residue could be accommodated in the crystal structure of α-Gal A, and that the number of main- and side-chain atoms that were influenced by this substitution was similar to other mutations known to produce later-onset forms of FD [13]. As this mutation appears to produce a functional enzyme, the reduction in its activity may result from instability or mis-targeting of the enzyme.

Our results demonstrate the existence of a frequent *GLA *mutation, the R118C in unrelated patients with FD. In this study the R118C mutation represents 60% of total unrelated patients and the D313Y represents 20% of total unrelated patients. A similar R118C frequency (43%) and D313Y (30%) was observed in a preliminary screening study of FD in a cardiology risk of Spanish and Portuguese population (Gaspar and Sá Miranda unpublished data). Since the R118C mutated enzyme is characterized by a high residual α-Gal A activity, genotype analysis should be carried out in males whose mean α-Gal A activity is 20-50% of the control mean. We recommend that biochemical and genotypic assays should be used as complementary tests to identify patients with FD.

Description of the pedigrees of the patients with these mutations is underway for the seven families (two patients were related). Genetic counselling has been provided and, after pedigree analysis, diagnostic testing will be offered to family members who may be affected. The study of family members has been initiated allowing the identification of seven FD patients within four families.

The results of this study indicate that the frequency of FD in patients with renal disease may be underestimated so far. We therefore propose that FD should be considered a cause of unexplained features such as cardiomegaly, arrhythmia and hypohidrosis in patients with renal disease. Even if patients are receiving maintenance dialysis, anticipating and treating other manifestations of FD (e.g. cardiac involvement and cerebrovasculopathy) is important. Moreover, the identification of an index case allows the study of family members and eventually ways to the diagnose of presymptomatic FD patients. General physicians and cardiologists should consider the diagnosis of FD in patients with LVH, cardiomyopathy, and conduction abnormalities, including short PR intervals and inverted ST segments without evidence of infarction. Microalbuminuria, or frank proteinuria, isosthenuria, and/or decreasing renal function should alert physicians to consider FD in the differential diagnosis.

## Conclusions

In conclusion, using a combined biochemical and DNA analysis strategy, we identified seven unrelated patients with *GLA *alterations, five with causal mutations (three males and two females) and two presenting the pseudo-allele D313Y who were undergoing haemodialysis for end-stage renal disease without a diagnosis. According to our results R118C seems to be a frequent *GLA *mutation in FD representing 60% of total identified mutated alleles. The two frequent *GLA *alterations (R118C and the D313Y) identified are associated with a high residual α-Gal A activity, suggesting that hemizygous patients with FD cannot be identified only by enzymatic assay. Studies to further characterize these *GLA *alterations are ongoing to ascertain whether the mutated proteins are unstable or mis-targeted. Moreover, our results indicate that there may be a significant number of patients with end-stage renal disease who have undiagnosed FD. FD may therefore be more common in patients with renal disease than previously believed. Studies in other populations of patients showing possible manifestations of FD are needed to assess the frequency of the later-onset phenotypes, particularly because disease frequency may vary in different ethnic or demographic groups.

## Methods

### Patients

The whole project was submitted for approval to the Ethics Committee (IRB) of the Hospital Universitari de Girona Dr Trueta. The IRB evaluated the project and found it to be adjusted to the ethical norms. All Patients, males and females, undergoing dialysis with end-stage renal failure of uncertain aetiology were recruited at eight hospitals (including 17 haemodialysis centres) from several regions of Spain (Figure 3). Male and female patients aged over 18 years undergoing dialysis were screened for FD. Patients provided written informed consent to participate in the study, which complied with the terms of the Declaration of Helsinki. The healthy subjects who underwent genetic screening were volunteers and gave formal informed consent for the DNA analysis.

### Assay for enzyme activity of α-Gal A

Screening for FD was carried out by measuring the activity of α-Gal A in DBS. Blood samples collected on Whatman number 903 paper (Whatman, Middlesex, United Kingdom) were kept at room temperature during shipment and stored at 4°C in zip-lock plastic bags. The name, sex, date of birth, hospital, physician and date of collection was recorded on the cards. DBS (diameter, 5 mm) were obtained from the sample card using a standard paper punch. DBS samples were added to 96-well micro titre plates and incubated overnight at 37°C with 4-methylumbelliferyl-α-D-galactopyranoside (Glycosynth, Warrington, UK) as a substrate, and N-acetyl-D-galactosamine (Sigma-Aldrich Quimica, S.A., Sintra, Portugal) as an inhibitor for α-N-acetylgalactosaminidase. Each plate contained an internal standard for quality-control purposes. The reaction was stopped with 250 μl Glycine-NaOH(pH 10.5). The concentration of hydrolysed 4-methylumbelliferyl (4-MU) was determined by measuring the fluorescence of the final mixture (excitation 365 nm; emission 450 nm) using a Cary Eclipse fluorescence spectrophotometer (Varian Incorporated, Walnut Creek, USA) and by comparison with 4-MU standards. Enzyme activities were expressed as nmol/h/spot.

### Molecular study

To identify patients with FD who exhibited α-Gal A residual activity, genotype analysis was carried out in all samples with enzyme activity lower than 80% or 40% of the control means in female and male patients, respectively. The upper limit in the case of the females was chosen accordingly to the enzymatic activity present in several heterozygous Fabry patients previously identified by us and used to validate this strategy. It is important to clarify that the identification of heterozygous for FD must always be based on genotype analysis. So, in this study, due to logistic reasons we can not rule out that some heterozygous could remain unidentified. DHPLC analysis of polymerase chain reaction (PCR) products was performed, as previously described [25], in order to identify genotypic alterations. In the case of exons that showed variations in their DHPLC profiles, the introns-exons boundaries were amplified by PCR [25]. The resulting products were purified using QIAquick PCR Purification kit (Qiagen, Izasa, Portugal) and the samples were sent to MWG Biotech (Fraunhoferst, Germany) for sequencing. Samples from 120 healthy individuals without FD were used as controls. RFLP (restriction fragment length polymorphism) analysis for the frequent mutation R118C was performed using *HinfI *(Fermentas, Ontario, Canada) with a final concentration of 1 U/μl and a final volume of 12 μl for 150 min at 37°C. A mutation was considered to cause disease if there was co-segregation of the mutation and the disease phenotype, or if there was low α-Gal A activity in the affected family.

## Competing interests

The authors declare that they have no competing interests.

## Authors' contributions

PG carried out the molecular and biochemical study as well as the drafting of the article. JH was the coordinator of the Spanish centers involved in the study. DR performed biochemical assays. SC, RD, CFA, RF, JM, MDP, MDP, PRA, GT and PV were involved in the collection and selection of the studied samples. MCSM carried out the conception and design of the study as well as the analysis and interpretation of the data. All the authors read and approved the final manuscript.

## Pre-publication history

The pre-publication history for this paper can be accessed here:

http://www.biomedcentral.com/1471-2350/11/19/prepub
